# Gastrodin inhibits cell proliferation in vascular smooth muscle cells and attenuates neointima formation *in vivo*

**DOI:** 10.3892/ijmm.2012.1100

**Published:** 2012-08-20

**Authors:** LIHUA ZHU, HONGJING GUAN, CHANGPING CUI, SONG TIAN, DA YANG, XINAN WANG, SHUMING ZHANG, LANG WANG, HONG JIANG

**Affiliations:** 1 Department of Cardiology, Renmin Hospital of Wuhan University, Wuhan;; 2 Cardiovascular Research Institute of Wuhan University, Wuhan, Hubei 430060, P.R. China

**Keywords:** gastrodin, vascular smooth muscle cell, neointima formation, platelet-derived growth factor

## Abstract

Vascular smooth muscle cell (VSMC) proliferation plays a critical role in the development of vascular diseases. In the present study, we tested the efficacy and the mechanisms of action of gastrodin, a bioactive component of the Chinese herb *Gastrodia elata* Bl, in relation to platelet-derived growth factor-BB (PDGF-BB)-dependent cell proliferation and neointima formation after acute vascular injury. Cell experiments were performed with VSMCs isolated from rat aortas. WST and BrdU incorporation assays were used to evaluate VSMC proliferation. Eight-week-old C57BL/6 mice were used for the animal experiments. Gastrodin (150 mg/kg/day) was administered in the animal chow for 14 days, and the mice were subjected to wire injury of the left carotid artery. Our data demonstrated that gastrodin attenuated the VSMC proliferation induced by PDGF-BB, as assessed by WST assay and BrdU incorporation. Gastrodin influenced the S-phase entry of VSMCs and stabilised p27Kip1 expression. In addition, pre-incubation with sinomenine prior to PDGF-BB stimulation led to increased smooth muscle-specific gene expression, thereby inhibiting VSMC dedifferentiation. Gastrodin treatment also reduced the intimal area and the number of PCNA-positive cells. Furthermore, PDGF-BB-induced phosphorylation of ERK1/2, p38 MAPK, Akt and GSK3β was suppressed by gastrodin. Our results suggest that gastrodin can inhibit VSMC proliferation and attenuate neointimal hyperplasia in response to vascular injury. Furthermore, the ERK1/2, p38 MAPK and Akt/GSK3β signalling pathways were found to be involved in the effects of gastrodin.

## Introduction

Vascular smooth muscle cell (VSMC) proliferation plays a key role in the pathogenesis of vascular diseases, including arteriosclerosis and restenosis after vein grafting or coronary intervention (1–3). Consequently, antiproliferative strategies have been employed to successfully prevent the development of vascular proliferative disease. Injury causes the release of multiple growth factors and cytokines that stimulate cell proliferation via multiple signalling mechanisms (4). Following angioplasty, platelet-derived growth factor (PDGF) has been shown to be an important signal that contributes to the initiation of the early cellular response to injury. Therefore, the identification of novel compounds that inhibit PDGF-dependent cell proliferation have the potential to improve existing therapeutic strategies and limit late cardiovascular complications, such as in-stent restenosis and bypass graft failure (5).

*Gastrodia elata* Bl, a traditional herbal medicine, has been widely used in China and Japan for thousands of years (6). Gastrodin (p-hydroxymethylphenyl-β-D-glucopyranoside) is the main bioactive component of *Gastrodia elata* Bl, and has been widely used clinically for the treatment of neurasthenia, dizziness, epilepsy, migraine, headache and dementia. Previous studies have shown that gastrodin has neuroprotective pharmacological effects (7). Gastrodin protects against hypoxia-induced toxicity in primary cultures of rat cortical neurons (8), rescues impairments of synaptic plasticity induced by lead in the rat hippocampus (9), suppresses the accumulation of calcium in PC12 cells induced by high glucose treatment and decreases cell apoptosis in the PC12 cell line following I/R injury *in vitro* (10), and it improves learning behaviour in a rat model of Alzheimer’s disease induced by intra-hippocampal Aβ 1–40 injection (11). However, little is known about the effects of gastrodin on cardiovascular diseases and neointima formation, and the workings of the related signalling mechanisms remain unclear. Therefore, we addressed whether gastrodin attenuates VSMC proliferation induced by PDGF-BB *in vitro* and/or neointima formation in the carotid artery following wire injury *in vivo*.

## Materials and methods

### Materials

Gastrodin was purchased from Sigma-Aldrich (St. Louis, MO, USA). Recombinant mouse PDGF-BB was purchased from ProSpec (Rehovot, Israel). The antibodies used to detect the total levels and the phosphorylation of ERK1/2, p38, AKT, GSK-3β and STAT3 were obtained from Cell Signaling Technology (Danvers, MA, USA). Antibodies specific for p27Kip1, glyceraldehyde-3-phosphate dehydrogenase (GAPDH) and proliferating cell nuclear antigen (PCNA) were also purchased from Cell Signaling Technology. Anti-SM22α and anti-smooth muscle-α-actin (SMA) were purchased from Abcam (Cambridge, MA, USA). Anti-desmin and anti-smoothelin were purchased from Santa Cruz Biotechnology, Inc. (Santa Cruz, CA, USA). Complete protease inhibitor, PhosSTOP, Cell Proliferation ELISA, 5-bromo-2′-deoxyuridine (BrdU) (colorimetric) and Cell Proliferation Reagent WST-1 kits were purchased from Roche Diagnostics GmbH (Mannheim, Germany). Other reagents were purchased from Sigma-Aldrich, except where specified. For the *in vitro* studies, gastrodin was dissolved in phosphate-buffered saline (PBS), and PBS alone served as a control.

### Cell culture

Rat VSMCs were isolated enzymatically from the thoracic aortas of Sprague-Dawley rats. These cells were cultured in DMEM/F12 medium containing 10% fetal bovine serum and were identified as VSMCs by smooth muscle-α-actin (SMA) immunostaining, as previously described (12,13). VSMCs were grown to 60–80% confluence and were serum-starved for 24 h. Three independent experiments were analysed for all data shown. VSMCs from passages 5 to 12 were used for the experiments in this study.

### Cell proliferation and DNA synthesis assay

VSMCs (5×10^3^/well) were seeded in a 96-well microplate, grown to 60% confluence and serum-starved for 24 h. Following preincubation with gastrodin for 1 h, the cells were treated with PDGF-BB (20 ng/ml) for 48 h. Cell proliferation and DNA synthesis were assessed using commercial non-radioactive colorimetric WST-1 and BrdU incorporation assay kits (Roche) according to the manufacturer’s instructions. The cell proliferation reagent WST-1 was used to measure the accumulation of the number of viable VSMCs based on the cleavage of tetrazolium salts incubated in the culture medium. DNA synthesis in VSMCs was assessed by the incorporation of BrdU.

### Flow cytometric analysis of cell cycle distribution

Cells were incubated with propidium iodide (PI) staining buffer and were then analysed using a flow cytometer (FACScan; BD Biosciences, Franklin Lakes, NJ, USA). G0/G1, S and G2/M cell percentages were counted using the Multicycle AV software (Phoenix Flow Systems, San Diego, CA, USA).

### Western blotting

VSMCs were treated with gastrodin (200 μg/ml) for 2 h prior to incubation with 20 ng/ml PDGF-BB for the indicated time. The VSMCs were lysed in RIPA buffer with a protease cocktail and a phosphatase cocktail (Roche). Cell extracts were used for SDS-PAGE and were then transferred to Immobilon-FL transfer membranes (Millipore) and probed with various antibodies. The protein bands were then incubated with a secondary IRDye^®^ 800CW-conjugated antibody and detected with an Odyssey Imaging System.

### Carotid artery wire injury model

All animal experimental protocols were performed according to institutional guidelines on animal welfare and were approved by the Ethics Committee at Renmin Hospital of Wuhan University. Eight-week-old male C57/BL6 mice were fed normal rodent chow or chow containing 0.09% gastrodin (w/w) for 14 days prior to wire injury. With this chow, the mice were fed ∼150 mg of gastrodin/kg/day. For the wire injury surgery, the mice were anesthetised with an intraperitoneal injection of sodium pentobarbital (90 mg/kg). The left carotid artery was exposed and looped on the external branch using an 8-0 silk suture. An angiotomy was performed in the internal carotid artery, and a straight metal guide wire (0.38 mm in diameter, C-SF-15-15; Cook, Bloomington, IN, USA) was inserted toward the aortic arch and then passed forward and withdrawn 5 times with a rotating motion, as previously described (14). The contralateral carotid artery served as a control. The mice were maintained on their respective chow until they were euthanised and processed for morphological studies at the indicated time points following the initial surgery.

### Histological and morphometric analyses

The carotid arteries were harvested 28 days post-injury. The animals were euthanised with an intraperitoneal injection of excessive sodium pentobarbital. Subsequently, the arteries were removed, fixed with 4% paraformaldehyde in PBS for at least 16 h and paraffin-embedded without further dissection. Serial sections (5 μm) were created across the site of injury, ∼300 μm from the branches of the left carotid artery. The areas of the intima and media were measured in H&E-stained sections in a blinded manner by a single observer using Image-Pro Plus 6.0 software (Media Cybernetics). A mean value was determined after assessing at least 4 sections from each mouse. Neointima formation was defined as the ratio of the intimal area to that of the medial area (I/M). For the PCNA immunohistochemical analysis, the sections were preincubated with 5% normal goat serum and then incubated with anti-PCNA monoclonal antibody (1:100, no. 2586, CST). The sections were then incubated with a biotinylated secondary streptavidin-horse-radish peroxidase antibody followed by diaminobenzidine (DAB kit; Dako), after which the sections were counterstained with haematoxylin. For the negative controls, the primary antibody was replaced with PBS. The mean value from at least 3 sections/mouse was used to quantitatively represent the number of PCNA-positive cells that were present in the injured vessel walls.

### Statistical analysis

The results were expressed as mean ± SEM. Statistical analysis was performed by one-way analysis of variance (ANOVA), followed by Dunnett’s multiple comparison tests. A P-value <0.05 was considered to indicate statistically significant differences.

## Results

### Role of gastrodin in VSMC proliferation

**Figure 1.**
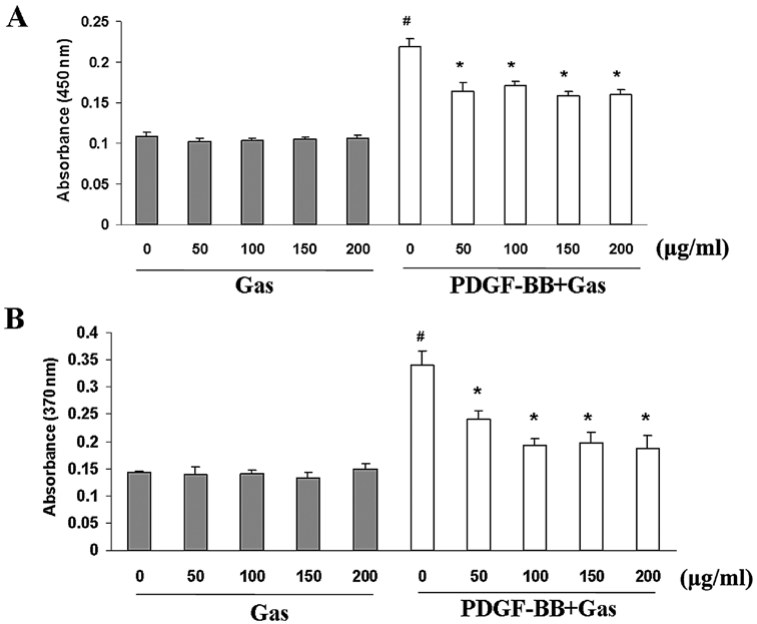
Gastrodin treatment prevents VSMC proliferation and DNA synthesis induced by PDGF-BB stimulation. VSMCs were treated with the indicated concentrations of gastrodin (50–200 μg/ml) for 48 h in the absence or presence of PDGF-BB (20 ng/ml). (A) Cell viability was examined with the WST test. Data are expressed as the mean OD450 ± SEM (^#^P<0.01 vs. control group; ^*^P<0.01 vs. PDGF alone; n=6). (B) BrdU incorporation was determined with an ELISA-based assay. DNA synthesis is expressed as the mean OD370 ± SEM (^#^P<0.01 vs. control group; ^*^P<0.01 vs. PDGF alone; n=6).

We first investigated the effect of gastrodin on proliferation using a WST-1 cell proliferation assay. Gastrodin and PDGF-BB were dissolved in serum-free medium for these experiments. Quiescent cells were pre-treated with gastrodin (50–200 μg/ml; Sigma-Aldrich) for 1 h prior to PDGF-BB stimulation (20 ng/ml; ProSpec). PDGF-BB stimulation increased VSMC proliferation by ∼2.5-fold compared with the control group at 48 h. Gastrodin markedly inhibited VSMC proliferation in a dose-dependent manner (Fig. 1A). BrdU incorporation assays were used to further investigate the effects of gastrodin on DNA synthesis. Gastrodin significantly inhibited the BrdU incorporation induced by PDGF-BB stimulation in a dose-dependent manner (Fig. 1B). Moreover, gastrodin treatment in the absence of PDGF-BB did not decrease the viability of the VSMCs or their incorporation of BrdU compared with the control cells, indicating that gastrodin treatment at these concentrations has no cytotoxic effects on VSMCs, and that the inhibitory effect of gastrodin targets DNA synthesis rather than cytotoxicity to cause the loss of cellular DNA (Fig. 1A).

### Role of gastrodin in cell cycle progression

**Figure 2.**
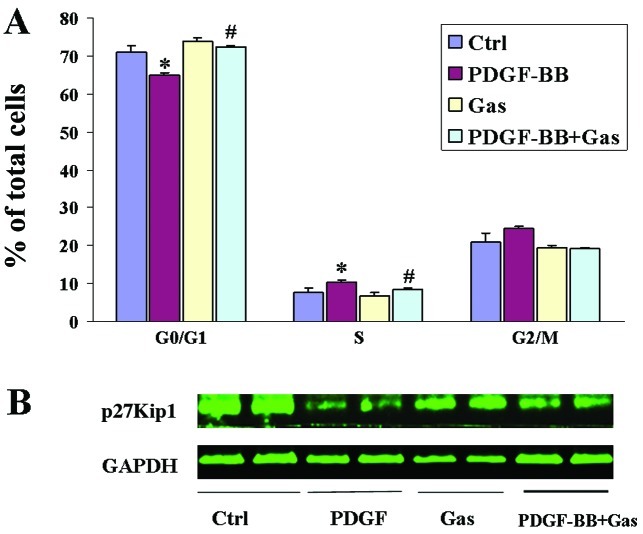
Gastrodin treatment prevents cell cycle progression in VSMCs. VSMCs were grown with gastrodin (200 μg/ml) in the absence or presence of PDGF-BB (20 ng/ml) for 24 h, and their cell cycle stage distribution was evaluated by flow cytometry. (A) Quantification of VSMCs in the G0/G1, S and G2/M phases according to flow cytometric evaluation (^*^P<0.05 vs. control group; ^#^P<0.05 vs. PDGF alone; n=3). (B) The protein levels of p27Kip1 were determined by western blot analysis.

Cell proliferation is controlled by progression through the cell cycle, which is regulated by many proliferative signalling cascades. Progression through the cell cycle requires the activation of cyclin/cyclin-dependent protein kinase (CDK) complexes, which are regulated by CDK inhibitory proteins (CKIs) such as p27Kip1. We analysed the effects of gastrodin treatment on the cell cycle stage distribution of VSMCs. As shown by flow cytometry, PDGF-BB stimulation significantly increased the percentage of cells in S-phase and decreased the percentage of those in the G0/G1 phases, whereas gastrodin (at a concentration of 200 μg/ml) significantly decreased the number of S-phase cells and increased the fraction of G0/G1-phase cells among the VSMCs (Fig. 2A). These data indicate that gastrodin can prevent S-phase entry in VSMCs. We next analysed the expression of p27Kip1 by western blotting. p27Kip1 was found to be constitutively expressed in quiescent VSMCs, and its expression was downregulated following PDGF-BB stimulation. By contrast, pretreatment with gastrodin markedly restored p27Kip1 expression (Fig. 2B).

### Role of gastrodin in VSMC phenotype switching

**Figure 3.**
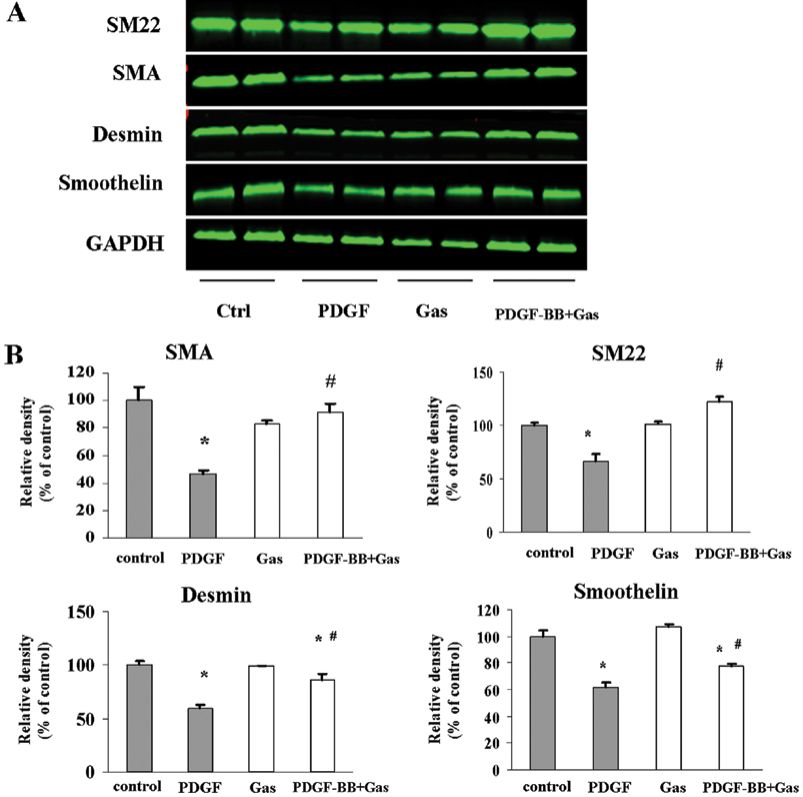
Effects of gastrodin treatment on the regulation of smooth muscle gene expression. (A) VSMCs were preincubated for 2 h with gastrodin (200 μg/ml) and then stimulated with PDGF (20 ng/ml) for 48 h. The protein levels of SMA, SM22α, smoothelin and desmin were determined by western blot analysis and quantified by densitometry. (B) Bar graphs showing the quantification of the western blot analysis results. The results are expressed as a percentage of the controls (^*^P<0.05 vs. control group; ^#^P<0.05 vs. PDGF alone).

In response to the vascular injury environment, VSMCs can dedifferentiate into a proliferative phenotype that is characterised by increased proliferation and decreased expression of smooth muscle markers, such as SMA, SM22α, desmin and smoothelin. Thus, we examined whether gastrodin modulates the phenotype of VSMCs in culture. Following pretreatment with gastrodin (200 μg/ml) for 2 h, quiescent VSMCs were stimulated with PDGF (20 ng/ml) for 48 h in the presence/absence of gastrodin. Western blotting was performed to detect the expression of SMA, smoothelin, SM22α and desmin. PDGF-BB stimulation reduced the protein levels of SMA, SM22α and desmin (Fig. 3). However, pretreatment with gastrodin reversed the repressive effect of PDGF-BB stimulation on the expression of SM-α-actin, smoothelin and desmin. These data indicate that gastrodin inhibits the ability of VSMCs to switch into the proliferative phenotype *in vitro*.

### Role of gastrodin in signalling pathways induced by PDGF-BB

**Figure 4.**
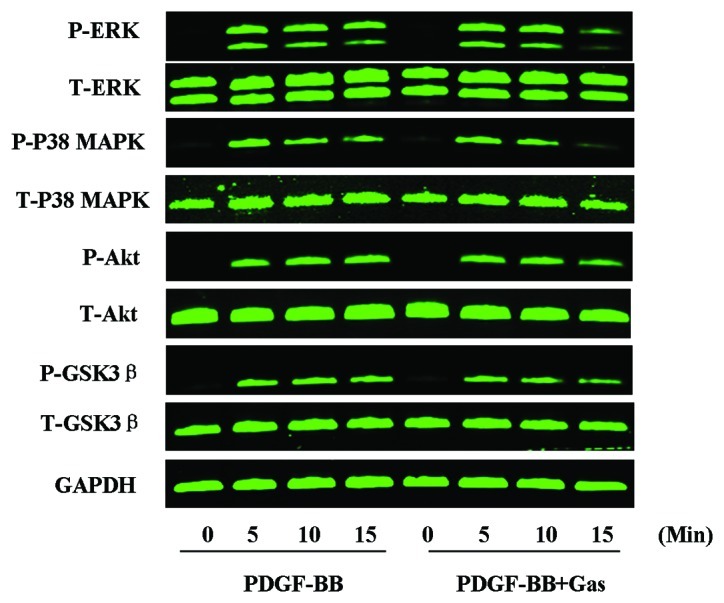
Inhibitory effects of gastrodin treatment on MAPK, Akt/GSK-3β and STAT3 activation in PDGF-BB-stimulated VSMCs. Serum-starved VSMCs were stimulated with PDGF-BB (20 ng/ml) for the indicated time in the absence or presence of gastrodin (200 μg/ml). The protein levels of phospho-ERK1/2, ERK, phospho-p38 MAPK, p38 MAPK, phospho-Akt, Akt, phospho-GSK-3β, GSK-3β were determined by western blot analysis. One representative image from 3 independently performed experiments is shown.

We next examined the signalling pathway(s) involved in the inhibitory effect of gastrodin on VSMC proliferation in response to PDGF-BB stimulation. ERK1/2, p38 MAPK, Akt and GSK3β phosphorylation were each increased at 5 min post-PDGF-BB treatment, and this effect persisted until 15 min post-PDGF-BB treatment. Whereas PDGF-BB stimulation induced the phosphorylation of ERK1/2, p38 MAPK, Akt and GSK3β, these modifications were significantly inhibited by gastrodin at 15 min post-treatment. The total protein levels of these signalling molecules did not change significantly during the course of stimulation with PDGF-BB in the presence or absence of gastrodin (Fig. 4). These data suggest that the MAPK and Akt signalling pathways are involved in the suppressive effects of gastrodin on the proliferation of VSMCs in response to PDGF-BB.

### Role of gastrodin in neointima formation and cell proliferation in vivo

**Figure 5.**
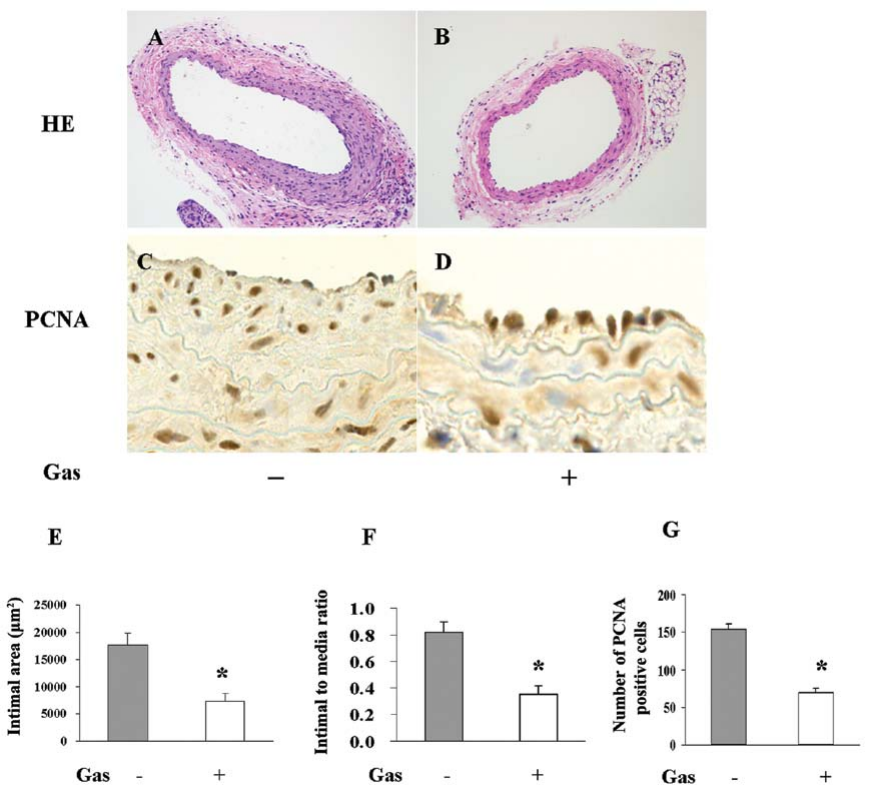
Gastrodin feeding prevents neointima formation induced by guide wire injury. (A and B) Representative H&E staining and (C and D) PCNA immunohistochemical staining images of the injured carotid arteries from either control-fed mice or gastrodin chow-fed mice. Quantification of the (E) intimal area, (F) I/M ratio and (G) PCNA-positive cells in the carotid arteries of animals from either the control group or the gastrodin-treated group (n=9; ^*^P<0.01 vs. injured control).

We observed that gastrodin could inhibit the proliferation of VSMCs, influence their cell cycle progression, reverse their phenotypic switch and suppress the signalling pathways induced by PDGF-BB. These findings suggest a potent therapeutic application for gastrodin against neointima formation in response to injury. To confirm this hypothesis, the left carotid arteries of mice were injured with a metal guide wire to induce a model of neointima hyperplasia. Neointima thickening 28 days following wire injury was reduced by 50% in the gastrodin chow-fed group compared to the normal chow-fed group. VSMC proliferation *in vivo* was characterised according to immunohistochemical staining with a PCNA antibody. Gastrodin chow consumption significantly diminished the proliferation response induced by arterial injury, as the number of PCNA-positive cells was more than 2-fold greater (P<0.01) in the normal chow-fed group than in the gastrodin chow-fed group (Fig. 5).

## Discussion

The present study demonstrates for the first time that gastrodin treatment inhibits VSMC proliferation induced by PDGF-BB *in vitro* and markedly suppresses neointimal hyperplasia following wire injury *in vivo*. These protective effects may be associated with a wide spectrum of signalling pathways, including MAPKs and Akt/GSK3β. These observations suggest that gastrodin may have beneficial effects for protecting against the neointima formation associated with arteriosclerosis and restenosis following percutaneous coronary intervention (PCI) and vein grafting.

Gastrodin, the main bioactive component of *Gastrodia elata* Bl, has potent protective effects against the nervous system and has been commonly prescribed by Chinese practitioners for the treatment of neurasthenia, dizziness, epilepsy, migraine, headache and dementia. Although gastrodin treatment has been shown to have many neuroprotective pharmacological effects, little is known about the effects of gastrodin on vascular disease and cell proliferation. Luo *et al* (15) reported that gastrodin could inhibit VSMC proliferation *in vitro*, as VSMC numbers and H^3^-TdR incorporation decreased significantly in a dose-dependent manner following treatment with gastrodin or an injection with *Gastrodia elata* Bl. However, that report did not determine whether gastrodin could inhibit the VSMC proliferation induced by mitogens, which is an important component of the pathological process of various vascular diseases (5,16–18). In the current study, we demonstrated that gastrodin treatment suppressed the increased cell number and DNA incorporation resulting from PDGF-BB stimulation. Moreover, cell proliferation is controlled by a large number of signalling proteins that regulate the mitotic cell cycle. p27Kip1, a CDK inhibitory protein (CKI), plays a key role in controlling the cell cycle in response to various pathophysiological processes (19–21). In quiescent cells, p27Kip1 is highly translated and demonstrates stable expression. Upon mitogenic stimulation, p27Kip1 is rapidly downregulated, which enables the activation of CDK/cyclin complexes and the subsequent transcriptional activation of genes required for the G1/S transition and the initiation of DNA replication (20). However, the effect of gastrodin on the modulation of the cell cycle was not examined. In this study, we found that gastrodin can influence the G/S transition of the cell cycle and stabilise p27Kip1, which is degraded following PDGF-BB stimulation. In addition to acting as a VSMC mitogen, PDGF-BB is a potent negative regulator of VSMC differentiation. VSMCs are remarkably plastic in response to the local environmental changes induced by vascular injury (22). The dedifferentiation of VSMCs is associated with dramatically increased cell proliferation, migration and synthetic capacity, and it plays a critical role in neointimal hyperplasia. In our study, we found that gastrodin treatment could restore the suppression of smooth muscle (SM)-marker gene expression induced by PDGF-BB, indicating that gastrodin significantly affects the manifestation of the VSMC phenotype *in vitro*.

The mechanisms by which gastrodin exerts its anti-proliferative effects remain unclear. Dai *et al* (7) reported that gastrodin could inhibit the expression of inducible NO synthase, cyclooxygenase-2 and proinflammatory cytokines in cultured LPS-stimulated microglia via the MAPK pathways. MAPKs have been shown to play important roles in PDGF-BB-induced cell proliferation in many cell types (23). It has also been reported that PDGF-BB induces the phosphorylation of Elk-1 through ERK1/2, and that phosphorylated Elk-1 competes with SRF for binding to CArG elements within the promoters of SMC marker genes, such as SM22α and SMA (24). Therefore, we investigated the effect of gastrodin treatment on the phosphorylation of three MAPKs induced by PDGF-BB stimulation in VSMCs. We observed that the phosphorylation of ERK1/2 and p38 was rapidly and markedly induced in VSMCs following PDGF-BB stimulation, whereas gastrodin pretreatment suppressed these effects, indicating that the inhibition of MAPK signalling is involved in the anti-proliferative effects of gastrodin.

Previous studies have demonstrated that growth factors such as PDGF-BB facilitate the phosphorylation of Akt in VSMCs. Moreover, Akt signalling regulates cell survival and proliferation in a variety of cell types (25,26). Accordingly, the transfection of a dominant-negative Akt (DN-Akt) mutant was found to attenuate hyperplasia through the inhibition of VSMC proliferation and the reversal of VSMC dedifferentiation (27,28). It is well known that GSK3β is an Akt substrate and that activated Akt can phosphorylate GSK3β and decrease its catalytic activity (29). GSK3β phosphorylates cyclin D1 at T286, which directs its proteolytic degradation. The catalytic activity of GSK3β is inhibited by Akt-dependent phosphorylation, and Akt-mediated GSK3β phosphorylation stabilises cyclin D1 (30). Akt activation also mediates the ubiquitination and degradation of p27Kip1 in response to mitogen stimulation via the upregulation of SKP2, a key component of the SCF^SKP2^ ubiquitin ligase complex. Moreover, Bonnet *et al* (31) demonstrated that the inhibition of Akt/GSK3β/NFAT signalling in response to PDGF could suppress VSMC proliferation and reverse injury-induced remodelling. In line with previous reports, we observed a marked increase in Akt activity and the phosphorylation of its substrate GSK3β in PDGF-BB-stimulated VSMCs. Furthermore, pretreatment with gastrodin facilitated the dephosphorylation of Akt and GSK3β, and stabilised p27Kip1. These results indicate that the Akt/GSK3β signalling pathway is partially involved in the inhibitory effects of gastrodin treatment on VSMC proliferation.

In conclusion, this study has clearly demonstrated that gastrodin treatment attenuates PDGF-BB-induced VSMC proliferation *in vitro* and injury-induced neointima formation *in vivo*. Gastrodin protects against VSMC proliferation by preventing the G/S cell cycle transition, suppressing the VSMC proliferative phenotypic switch and by inhibiting ERK1/2, p38 and Akt/GSK3β signalling. Thus, we believe that gastrodin is a strong candidate for effective therapy in the prevention of vascular proliferative disease.
